# Bacterial Succession in Microbial Biofilm as a Potential Indicator for Postmortem Submersion Interval Estimation

**DOI:** 10.3389/fmicb.2022.951707

**Published:** 2022-07-22

**Authors:** Finkelbergs Dmitrijs, Juanjuan Guo, Yecao Huang, Yafei Liu, Xinyue Fang, Kankan Jiang, Lagabaiyila Zha, Jifeng Cai, Xiaoliang Fu

**Affiliations:** ^1^Department of Forensic Science, School of Basic Medical Sciences, Central South University, Changsha, China; ^2^Department of Vascular Surgery, Shenzhen Second People's Hospital, The First Affiliated Hospital of Shenzhen University Health Science Center, Shenzhen, China; ^3^Shenzhen Institute of Advanced Technology, Chinese Academy of Sciences, Shenzhen, China; ^4^Department of Forensic Medicine, School of Basic Medical Sciences and Forensic Medicine, Hangzhou Medical College, Hangzhou, China

**Keywords:** postmortem interval, postmortem submersion interval, microbial biofilm, bacterial succession, machine learning algorithm

## Abstract

Bacteria acts as the main decomposer during the process of biodegradation by microbial communities in the ecosystem. Numerous studies have revealed the bacterial succession patterns during carcass decomposition in the terrestrial setting. The machine learning algorithm-generated models based on such temporal succession patterns have been developed for the postmortem interval (PMI) estimation. However, the bacterial succession that occurs on decomposing carcasses in the aquatic environment is poorly understood. In the forensic practice, the postmortem submersion interval (PMSI), which approximately equals to the PMI in most of the common drowning cases, has long been problematic to determine. In the present study, bacterial successions in the epinecrotic biofilm samples collected from the decomposing swine cadavers submerged in water were analyzed by sequencing the variable region 4 (V4) of 16S rDNA. The succession patterns between the repeated experimental settings were repeatable. Using the machine learning algorithm for establishing random forest (RF) models, the microbial community succession patterns in the epinecrotic biofilm samples taken during the 56-day winter trial and 21-day summer trial were determined to be used as the PMSI predictors with the mean absolute error (MAE) of 17.87 ± 2.48 ADD (≈1.3 day) and 20.59 ± 4.89 ADD (≈0.7 day), respectively. Significant differences were observed between the seasons and between the substrates. The data presented in this research suggested that the influences of the environmental factors and the aquatic bacterioplankton on succession patterns of the biofilm bacteria were of great significance. The related mechanisms of such influence need to be further studied and clarified in depth to consider epinecrotic biofilm as a reliable predictor in the forensic investigations.

## Introduction

During the forensic investigations, corpses can be found in a variety of natural or artificial aquatic environments, such as ponds, rivers, lakes, seas, and water storage containers. These cases may be suicides, homicides, or accidents, with the most common death cause being asphyxiation by drowning in the water. Due to the lower temperature and the oxygen-deficit, the postmortem decomposition in the aquatic environments is usually slower than that on the land, which makes the forensic identification of corpses in the water particular. Postmortem submersion interval (PMSI), which is defined as the period between the entry into the water and recovery of the dead body, is approximately equal to the postmortem interval (PMI) in most of the common cases of drowning (Humphreys et al., 2013). It can be used for inferring the location of body falling into the water, delimiting the scope of the search for the suspect, and providing direction for the investigation. For corpses found on land, the PMI estimation relies on the temporal changes of the postmortem decomposition. However, determining PMSI has long been problematic because the postmortem decomposition in water is affected by a variety of biotic and abiotic factors, such as microbial metabolism, algal growth, and adipocere formation (Haefner et al., 2004; Pakosh and Rogers, 2009; Widya et al., 2012; Ueland et al., 2014). For example, corpses submerged in freshwater (rivers) may show more obvious decomposition changes than those submerged in saltwater (seas), as lower temperatures and higher salinity slow down bacterial activity (Byard, 2018). It is evident that microbial metabolism is the leading factor affacting the rate of postmortem decomposition happening in the terrestrial environment (Lauber et al., 2014; Metcalf et al., 2016). Based on this, several studies have monitored the bacterial community successions on the corpses found on land, and suggested that the postmortem changes in microbial communities were dramatic, measurable, and repeatable, allowing PMI to be estimated accurately even within a long time frame (Metcalf et al., 2013, 2016; Burcham et al., 2019). However, such estimation is more complicatied for carcasses found in water, especially for the cases of drowning. When drowning occurs, aquatic microorganisms will enter the respiratory and digestive tracts along with the liquid, changing the structure of endogenous microbial communities, consequently affecting their succession patterns. Moreover, if any unpredictable rupture occurs on the decomposing carcass, fluid from the environment could also enter the body and interrupt previous succession processes. All these complex scenarios are hard to evaluate in the forensic practices. As a result, to this date, limitied research has been done on the succession of the microbial communities on carcasses found in water.

When free-swimming aquatic microbes encounter solid surfaces, they can gradually switch from a planktonic lifestyle to forming biofilms that are wrapped by the extracellular polymeric substance (EPS) (Battin et al., 2007). EPS contributes to plankton attachment and provides independent niches specific to the particular aquatic environments. The human corpse appearing in the water provides attachment surface and organic nutrients for the aquatic microbes to form specific epinecrotic biofilm. A growing body of excellent studies has been done on biofilms in multiple disciplines, such as food hygiology (Liu et al., 2015), ecology (Ram et al., 2005), and medicine (Huang et al., 2011; Johnson et al., 2015), but there is limited data available associated with the human corpse decomposition in the water. The bacterial succession patterns of the epinecrotic biofilms have been studied by several forensic research groups using surrogate models of the vertebrate corpses. Dickson et al. were the first to evaluate the potential use of the bacterial succession for the PMSI estimation (Dickson et al., 2011). They have investigated the microbes involved in decomposition of porcine cadavers in the sea and confirmed that marine bacteria rapidly colonized the skin of the submerged cadavers in a succession manner. Using high-throughput sequencing (HTS), Benbow et al. have provided the first metagenomic data which has described aquatic bacteria succession patterns in the epinecrotic biofilms on the porcine remains submerged in a freshwater habitat (Benbow et al., 2015). The biofilm communities on the submerged remains changed significantly through the PMSI. Although the microbial community differences between the summer and winter trials have been observed, the succession patterns of dominant phyla and genera were similar. In recent years, machine learning (Li et al., 2019), and even deep learning algorithms (Rahaman et al., 2020), have been applied in the medical field on a large scale, as a result providing new tools for forensic research to explore microbial succession patterns. Using replicate swine carcasses, Kaszubinski et al. (2020) performed an experiment to describe the variation of epinecrotic biofilm microbiome in a non-flowing aquatic habitat. Through the sufficient sequencing data obtained, Kaszubinski et al. modeled key taxa for estimating PMSIs using the machine learning algorithm (random forest (RF) regression), correctly estimating the PMSI ± 3 days with *R*^2^ = 97.50%. These studies have provided compelling evidence that the bacterial succession in the epinecrotic biofilm has a prominent potential to be used for the PMSI estimation in forensic investigations of submerged corpses.

To further the understanding of the biofilm succession in our geographical region and its potential to be used as an indicator in the PMSI estimation during forensic investigations, present study used juvenile swine as proxies for human corpses to establish decomposition models for the use in a non-flowing aquatic environment. Sterile tiles were used as the inorganic solids placed in both close and distant proximity from the decomposing carcasses for the epilithic biofilm attachment. We employed HTS to fully describe the bacterial successions in the epinecrotic biofilms on the decomposing carcasses and epilithic biofilms on the submerged tiles. The objectives of the research were to (1) characterize the succession patterns of the bacterial communities in the epinecrotic and epilithic biofilms; (2) assess the repeatability of the succession patterns in the replicable settings; (3) compare the differences in succession patterns between the summer and winter trials; and (4) seek to provide important data for developing a machine learning algorithm to estimate PMSI.

## Materials and Methods

### Study Design

**Figure 1:**
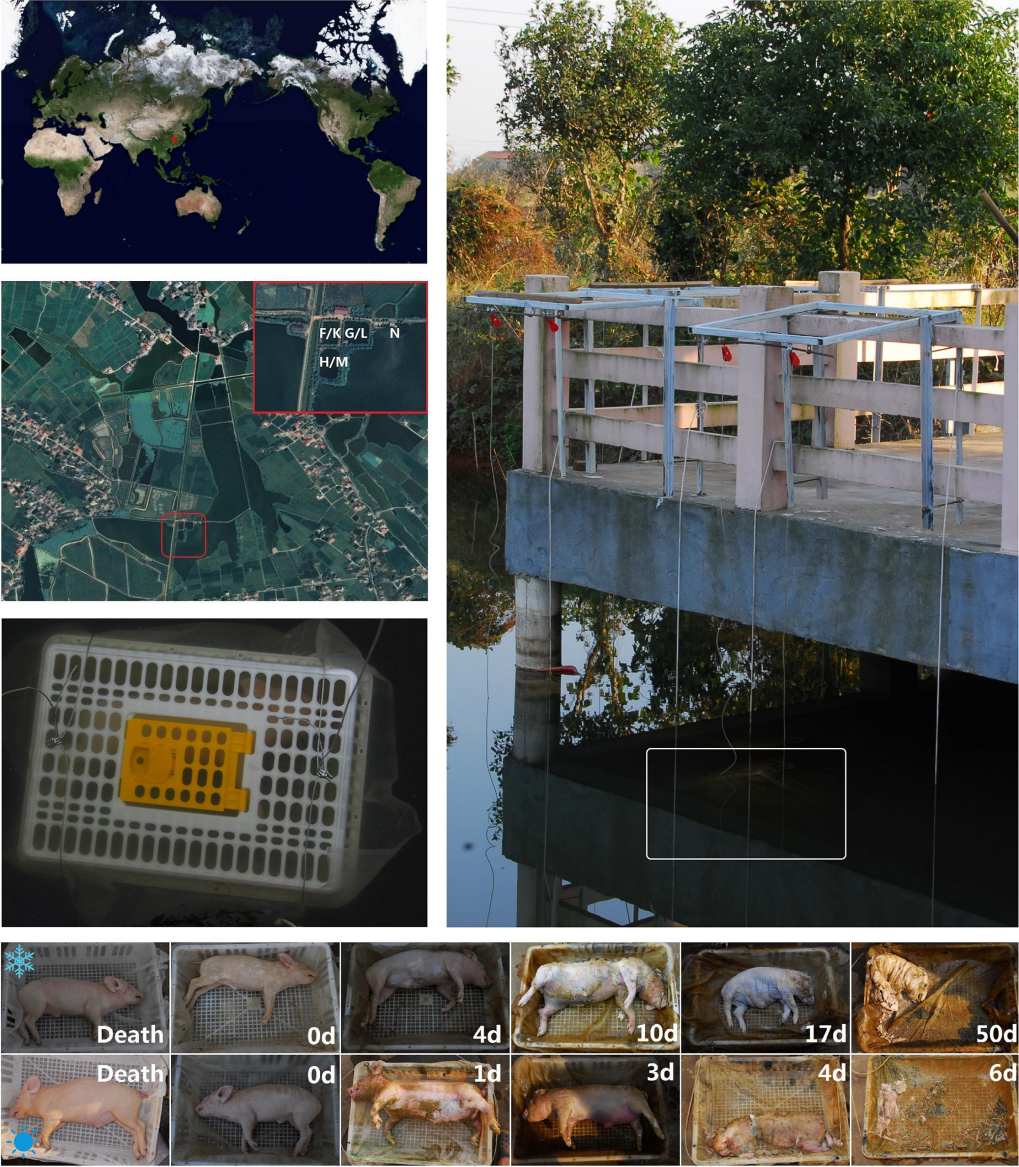
The geographic position and experimental scenes. Experiments were conducted in the 3 adjacent freshwater ponds and a nearby vast lake in Changsha, China. The white letters labeled on the satellite photo represent the experimental sites for the winter trail (F, G, & H), summer (K, L, & M) trial and the negative control (N). Carcasses were individually placed inside the plastic cages and then sunk into the water. The bottom pictures are, respectively, taken from the time immediately after death, submerged fresh, early floating, floating decay, advanced floating decay, and sunken remains stages of the carcasses in the winter (above) and summer (below) trials. The time points were labeled on each picture.

This study was approved by the Medical Ethics Committee of Xiangya Hospital, Central South University (approval number: 201503465) and followed all applicable institutional and national guidelines for the care and use of animals. A total of two drowning experiments were, respectively, conducted from 31 October to 26 December 2017 (winter trial) and 21 July to 11 August 2018 (summer trial) in three adjacent freshwater ponds (28°34′74.6″ N, 112°81′95.5″ E) within the Xiangjiang River watershed in Changsha, China (Figure 1). Each of the ponds was about 1,000–1,500 m^2^ and 5 m deep, subjected to direct sunlight (no canopy), and surrounded by some shrubs. The water in the ponds was previously pumped from a vast lake nearby.

Swine carcasses have been frequently used as proxies for human corpses in the forensic research (Schoenly et al., 2007). In our experiments, six female swines (Sus scrofa demesticus, *n* = 3 per trial), each weighing 7.05–11.5 kg, were purchased from a local farm and killed by drowning after anesthesia. Carcasses were individually placed on a fine mesh nylon pad (60 mesh/inch) inside the plastic cages (0.75 × 0.55 × 0.25 m^3^) to facilitate weighing as the carcass disarticulated and to prevent removal of the carcasses by scavengers (e.g., fishes, shrimps, and crabs) (Figure 1). Moreover, bricks were attached to the bottom of the cages to prevent the carcasses emerging from the water during the bloated stage, consequently inhibiting colonization by terrestrial insects. Through these measures, the microbial biofilms could form naturally on the carcass surfaces without the random interference from aquatic and terrestrial scavengers. Each cage was placed in a single pond and about 1 m below the water surface. In order to explore the influence of carcass decomposition on the formation of epilithic biofilm, synchronous sampling of 2 tiles which were placed in the pond 0.5 m away from oppsite sides of each carcass was conducted. About 100 m from the location of sunken carcasses, another two sterile tiles were placed 1 m below the water surface in the nearby lake during the summer trial as a negative control. In the process of decomposition, the postmortem changes were recorded and photographed daily. To visually describe the decomposition processes of the carcasses, the duration of each decay stage was assessed using the framework of Zimmerman and Wallace (2008) and Wallace et al. (2008). The visual body score was evaluated using the total aquatic decomposition scoring (TADS) system as described by van Daalen et al. (2017).

### Environmental Parameters and Sample Collection

Air temperature and relative humidity were recorded hourly by a data logger (MEACON Automation Technology Co., Ltd., Hangzhou, China). Water quality parameters, including water temperature (°C), dissolved oxygen (DO, mg/L), pH, conductivity (mS/cm), and salinity (ppt), were measured 4 times every day at 1 m beside each carcass and at the location for the negative control using the AZ 86031 Water Quality Checker (AZ Instrument Co., Taiwan, China), with its probe been placed 1 m below the water surface. Water temperature data recorded every 6 h during each of the experiment days was used to calculate the average daily water temperature, and accumulated degree days (ADDs) were calculated by summing the average daily water temperature above the lower development threshold (LDT) (Mateus and Vieira, 2014). Because ADD was used to explore how microorganism communities (not insects) changed during the study, an LDT of 0°C was employed for this calculation, according to Pechal et al. (2014).

A total of three types of microbial specimens were collected: epinecrotic, epilithic, and aquatic samples. Swine skin has been widely used as a proxy for human skin (Sekkat et al., 2002). Epinecrotic samples were collected from the skins of swine carcasses at multiple time points: 10 min before being sacrificed, immediately after being sacrificed, daily in the first week, and then weekly in the following days during the summer trial. Due to the carcass decomposition progressing slowly at low temperatures, the sampling time points after the swine sacrifice for the winter trial were different: every 3 days in the first week, and then every 2 week in the following days. At each sampling time point, a 10 × 10 cm^2^ skin area on one side of the torso of each carcass was gently swabbed for 60 s using sterile cotton applicators with care taken to not duplicate any previously sampled area. Then, the tip of each applicator was cut off with a pair of sterile scissors and placed in a 1.5-ml microcentrifuge tube. Epilithic and aquatic samples were collected from the tiles and ponds weekly during the summer trial and biweekly during the winter trial. The epilithic communities on the surfaces of each tile were sampled in the same way as the epinecrotic samples. For sampling the aquatic communities, one liter of pond water was collected at 1 m distance from each carcass using the sterile syringes, consequently filtered by suction filtration using a Buchner funnel and the nylon filter membranes (pore diameter 0.2 μm). The negative control of the epilithic and aquatic samples were taken further away from the carcasses from the nearby lake during the summer trial. All samples were immediately frozen at −80°C until further processing. Carcasses and tiles were immediately submerged back into the water to their original location upon sample collection completion, while also making sure that the disposable sterile gloves used in the procedure were replaced after each sampling. The detailed information of each sample is shown in Supplementary Table S1.

### DNA Extraction and PCR Amplification

The cotton tip of each swab or the fragment cut from each filter membrane was put in a bead tube for genome DNA extraction. The succeeding steps were performed as dictated by the manufacturer's specifications of MoBio PowerSoil DNA Isolation Kit (Mo Bio Laboratories, Carlsbad, CA, USA). Swabs and filter membranes that had not been used for sampling were used as the blank controls. DNA concentration and purity were determined *via* Nanodrop ultraviolet spectrophotometric detection and 0.8% agarose gel electrophoresis (Supplementary Table S1). All the qualified DNA samples were diluted to a 20 ng/μl working stock using sterile ultrapure water. When insufficient, the stock solution was used directly.

The V4 region of the 16S rDNA gene has been amplified using dual-indexed primers (515F/806R: 5′-GTGYCAGCMGCCGCGGTAA-3′; 5′-GGACTACNVGGGTWTCTAAT-3′) as described previously (Claesson et al., 2010). Each of the forward primers contained a 6 bp barcode unique to each sample. All polymerase chain reactions (PCRs) were conducted in 25 μl reaction volumes containing 5 μl of 5× reaction buffer, 5 μl 5× GC buffer, 2 μl dNTP (2.5 mM), 1 μl forward and reverse primers (10 μm), 2 μl DNA template, 8.75 μl ddH_2_O, and 0.25 μl Q5 DNA polymerase. Thermal cycling conditions were following: initial denaturation at 98°C for 2 min, followed by 30 cycles of denaturation at 98°C for 15 s, annealing at 55°C for 30 s, and extension at 72°C for 30 s, with a final extension at 72°C for 5 min. The resulting amplicons were mixed with the same volume of 1× loading buffer (containing SYB green) and detected using electrophoresis on 2% agarose gels. Except for the blank controls which failed amplification, all amplicons were in the size range of 200–300 bp and excised from the agarose gel for further experiments.

### Amplicon Sequencing and Data Analysis

All amplicons in the size range of 200–300 bp were purified using the AxyPrep DNA Gel Extraction Kit (Axygen Biosciences, Santa Clara, CA, USA) and pooled into equal concentrations. Sequencing libraries were generated using the TruSeq^®^ Nano DNA LT Library Prep Kit (Illumina, San Diego, CA, USA) following the manufacturer's recommendations, and index codes were added. The library quality was assessed on the Agilent Bioanalyzer 2100 system using Agilent High Sensitivity DNA Kit (Agilent, Santa Clara, CA, USA) and the Promega QuantiFluor Fluorometer using Quant-iT PicoGreen dsDNA Assay Kit (Thermo Fisher Scientific, Carlsbad, CA, USA). Finally, the library was sequenced on an Illumina MiSeq platform, which generated 300 bp paired-end reads.

After sequencing, the paired-end reads were assigned to the samples based on their unique barcode, truncated by cutting off the barcode and primer sequence, and merged using FLASH (version 1.2.7) (Magoč and Salzberg, 2011). The merged reads containing ambiguous bases (*N*) or low-quality bases were filtered out using QIIME filter (version 1.8.0) (Bokulich et al., 2013), and chimeras were removed using USEARCH (version 5.2.236) (Edgar et al., 2011). After the removal of singleton sequences, operational taxonomic units (OTUs) were classified with the threshold of 97% similarity using the UCLUST in QIIME (Edgar, 2010). A representative sequence was picked by selecting the longest sequence that had the largest hit number to other sequences in each OTU. Representative sequences of 16S OTUs were, respectively, aligned and annotated using the Greengenes database (Release 13.8, http://greengenes.secondgenome.com/) (DeSantis et al., 2006). Total raw sequencing data was published in the Sequence Read Archive (SRA) under the accession number PRJNA841063.

### Statistical Analysis

To avoid biases of biodiversity data generated by the number of sequences, the data were rarefied to 90% of the minimum library size. Statistical analysis was conducted using a web-based tool, MicrobiomeAnalyst (Dhariwal et al., 2017). For the hierarchical cluster analysis (HCA), each OTU began as a separate cluster, then the clustering algorithm proceeded to combine them until all OTUs belonged to a single cluster. Distances between OTUs were measured with Minkowski and clustering algorithms using the average linkage (the distance between two clusters is the average of the distances between all the points in those clusters). The results were visualized as a heat map to show the temporal changes in the taxonomic clusters. The alpha diversity indices of Chao1 and Shannon were calculated to evaluate the species richness and evenness of a sample. The statistical significance of differences in alpha diversity indices between experimental groups/decay stages was estimated using the analysis of variance (ANOVA). Principal coordinate analysis (PCoA) using weighted UniFrac dissimilarities was performed to visualize the changes in the community beta diversity according to the decomposition progress/experimental grouping. The statistical significance of the clustering pattern in the ordination plot was evaluated by permutational multivariate analysis of variance (PERMANOVA). The taxonomic composition of each sample was visualized in a stacked bar plot by the chronological order. Linear discriminant analysis effect size (LEfSe) was used to identify taxa with significantly differential abundance across experimental groups. Random forest (RF) analysis, which is a powerful machine learning algorithm for the identification of the predictive biomarkers and establishment of the prediction the regression of model, has been employed for the regressing of the OTU relative abundances against the ADDs using the “randomForest” R package. The relative abundance of an OTU is its percentage in the total amount of sequences in a sample. The OTUs were ranked in the order of their feature importance and selected to generate predictive biomarker sets. The RF regression models were futher established for predicting the PMSI based on generated biomarker sets were further established. The mean absolute error (MAE) and goodness of fit (*R*^2^) were used to evaluate the performance of the models (Metcalf et al., 2013).

## Results

### Progression of Carcasses Decomposition

During the summer trial, the average air temperature and relative humidity were 34.40 ± 1.98°C and 34.19 ± 3.78%, respectively. The carcasses decomposed much faster than those during the winter trail and progressed to the stage of sunken remains in one week time (Figure 1). Sample collection was thus conducted on the bone remains once a week for the following 2 weeks. By visually describing the decomposition phenomenon progression of each carcass, decay stages were determined as follows: submerged fresh stage began at 0.0 ± 0.0 day, early floating stage at 1.0 ± 0.0 days, floating decay stage at 2.8 ± 0.8 days, advanced floating decay stage at 4.0 ± 0.0 days, and sunken remains stage at 6.0 ± 0.0 days. During the 8-week winter trial, the average air temperature and relative humidity were 15.00 ± 4.84°C and 81.50 ± 14.82%, respectively. The carcasses decomposed slowly and entered sunken remains stage in a gradual manner. The submerged fresh stage began at 0.0 ± 0.0 day, early floating stage at 4.0 ± 1.0 days, floating decay stage at 10.0 ± 0.0 days, advanced floating decay stage at 17.3 ± 2.3 days, and sunken remains stage at 47.3 ± 7.5 days.

Water quality parameters were measured at the locations of sunken carcasses and the negative control tiles. The changes of calculated average water temperature, DO, pH, conductivity, and salinity are shown in Supplementary Figure S1. The water temperature was mainly affected by the climate and remained relatively constant in the summer but dropped gradually in the winter. ADDs increased linearly during both seasons. DO was mainly affected by the weather and dropped markedly on the rainy and cloudy days, most likely due to the photosynthesis decline of the underwater plants rather than the carcass decomposition influence. The fluctuations of water DO, pH, conductivity, and salinity in the summer were greater than those during the winter. However, there was no significant difference of water quality between the locations whether the carcasses had presented. These measurements of water quality indicated that the carcasses which would randomly appear in the pond would not cause serious changes to an aquatic environment with a large storage capacity. This also indicates that the water in these conditions can provide a relatively stable physicochemical environment for the drowned carcasses.

The body weight and TADS score changes during the decomposition processes are shown in Supplementary Figure S2. The loss of body weight and the increase of TADS scores in each trial were continued. However, the carcasses that decomposed in the summer progressed to the sunken remains stage more rapidly and had made their weight and TADS scores reach the plateau much earlier. In fact, the hyper slow decaying carcasses during the winter trial remained amounts of soft tissue even after 56-day decomposition. It is difficult to accurately describe such a slow process in detail using the protocols described by van Daalen et al. (2017), which limits the TADS system use for the accurate PMSI estimation for the winter cases. On the other hand, due to the impact of the water flow, the linear relationships between the body weight and the ADD in both trials were not significant. These results suggested that it is difficult to determine temporal changes precisely through the decay phenomena and weight loss.

### Overview of Sequencing

A total of 131 samples had been collected, including 66 in the winter trial and 65 in the summer trial. The details of each sample are shown in Supplementary Table S1. After the DNA extraction and PCR amplification, a total of 5,816,237 high-quality reads were received from the output data of 16S amplicon sequencing, and 140,618 OTUs were obtained after these reads had been classified with 97% similarity. All the read lengths were distributed in 200–300 bp, which fits the size of the 16S V4 region (Supplementary Figure S3). Based on the sequencing data rarefying to 90% of the minimum library size, a total of 2,366,112 clean reads and 6,897 simplified OTUs were used for the following statistical analysis. The numbers of clean reads and simplified OTUs of each sample are shown in Supplementary Table S1. The average good's coverage value for all samples was 99.80 ± 0.14%, which suggested that the number of reads was enough to analyze biodiversity in all samples. The rarefaction curves indicated that the species richness in each sample had approached the plateau phase, and it was unlikely that more species would been detected with additional sequencing efforts (Supplementary Figure S4). After representative sequences of OTUs had been annotated in the Greengenes database, the taxon number at each taxonomic level of each sample type is shown in Supplementary Table S2. The taxa of epinecrotic, epilithic, and aquatic communities in the winter trial were significantly less than those in summer, especially the taxa at each taxonomic level in epinecrotic communities which were less than half of that in summer. These results suggested that the season could be the main factor affacting the aquatic environments, thus leading to the changes in the biofilm community structures on the carcasses in the water.

### Community Differences Between Epinecrotic and Epilithic Biofilms

The calculated alpha diversity indices of Chao1 and Shannon for each sample are shown in Supplementary Table S1. The statistical differences in alpha diversity indices between the epinecrotic, epilithic, and aquatic communities were estimated using the ANOVA and reviewed as box plots (Supplementary Figure S5). During the winter trial, there were significant differences between the alpha diversity indices of the three communities. Each index of epinecrotic community was lower than that in the epilithic community, especially for the late decay periods. However, a significant difference between the communities was only be found in Chao1 indices for the summer trial, which indicated that there was a large difference in species richness, but a small difference in species evenness among the three communities in summer.

**Figure 2:**
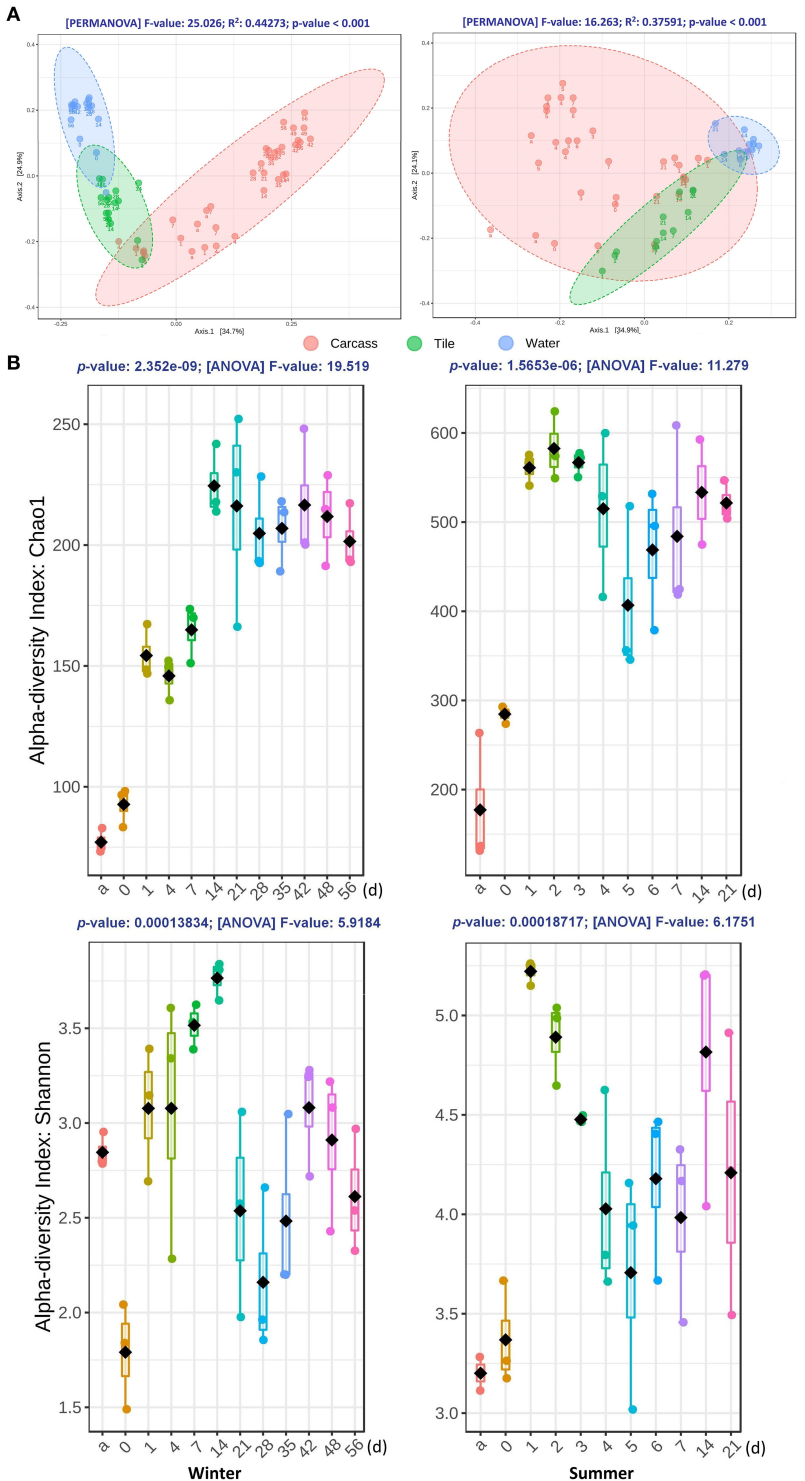
The diversity variations of the bacterial communities during the winter (left panel) and summer (right panel) trials. **(A)** Two-dimensional PCoA plots of weighted UniFrac distance matrices for samples obtained from carcasses, tiles, and water at different time points during both seasons. Samples obtained from different substrates are presented in different colors. Sampling time points are marked under each dot. The statistical significance of the clustering pattern in each plot was evaluated with PERMANOVA (top of each plot). **(B)** The alpha diversity variation of epinecrotic communities during the both seasons. Samples from each time point were measured with Chao1 and Shannon indices, as shown in each box plot. The *X*-axis of each plot are the sampling days and “a” represents the antemortem. The ANOVA results are showing at the top of each plot.

By measuring the weighted UniFrac distances, the beta diversity variation of the communities was visualized in PCoA plots and tested by PERMANOVA. As shown in Figure 2A, three types of samples were clustered into the significant clusters separately. The epilithic and aquatic community beta diversity was relatively stable during both seasons. Nevertheless, the epinecrotic communities showed a significant succession pattern through the time, which despite being initially close to the epilithic samples, subsequently separated from the other dots on the PCoA plots. Additionally, the communities present on the skeletal remains collected during the sunken remains stage (14 and 21 days) during the summer trial were also similar to the epilithic communities, indicating that the bones' adsorbability to the planktonic microorganisms was similar to that of the inorganic solid surface. Supplementary Figure S6 shows that there was a significant difference between the epilithic and aquatic community structures regardless of the presence of the decomposing carcasses. The epilithic samples from both the summer trial and negative control clustered together, while, aquatic samples formed into another cluster. Similar to the measurements of the water quality, these results suggested that the carcasses decomposing in the water had little impact on the aquatic environments and nearby epilithic biofilms.

According to the LEfSe analysis of the winter trial data, the phyla, such as Cyanobacteria, Actinobacteria, Planctomycetes, Fusobacteria, Chloroflexi, and Verrucomicrobia in the epinecrotic communities have significantly less presence than in the epilithic communities, whereas Firmicutes and Bacteroidetes were more present in the epinecrotic communities. As for the summer trial data analysis, Actinobacteria, Chloroflexi, Planctomycetes, and Verrucomicrobia in the epinecrotic communities were significantly less present, whereas Firmicutes were more present in the epinecrotic communities than in the epilithic communities. The main differentiated phyla between the epinecrotic and epilithic communities were similar during the two seasons (Supplementary Table S3). The differentiated genera of the two communities during two seasons are shown in Supplementary Table S4.

### Epinecrotic Community Temporal Variations During Carcasses Decomposition

The alpha diversity of epinecrotic communities changed significantly during both seasons (Figure 2B). For the winter trial, the Chao1 indices had increased after the carcasses entered the water (1 day) and during the floating decay stage (14–56 days), however, the Shannon indices had decreased immediately after death (0 day) and then increased after the carcasses entered the water (1–21 days). The alpha diversity for the summer trial followed a similar pattern. The Chao1 indices had increased after the carcasses entered the water (1 day) and only slightly reduced in the advanced floating decay stage (5–7 days). The Shannon indices had increased first after the carcasses entered the water (1 day), then decreased to a plateau (2–7 days), and finally slightly elevated during the sunken remains stage (14–21 days).

On the PCoA plot for the epinecrotic communities of both seasons (Supplementary Figure S7), all the dots of the summer samples clustered with the winter samples which were obtained before the 14 days of the decomposition. The dots of 21–56 days for the winter trial formed another cluster and separated from the previous samples. Supplementary Figure S8 illustrates that the seasonal factors had altered the beta diversity of aquatic communities. These results indicated that the beta diversity variation in epinecrotic communities during the winter was much more significant than that in summer, which could be attributed to the specific aquatic community structure and longer decomposition processes during the winter trial.

**Figure 3:**
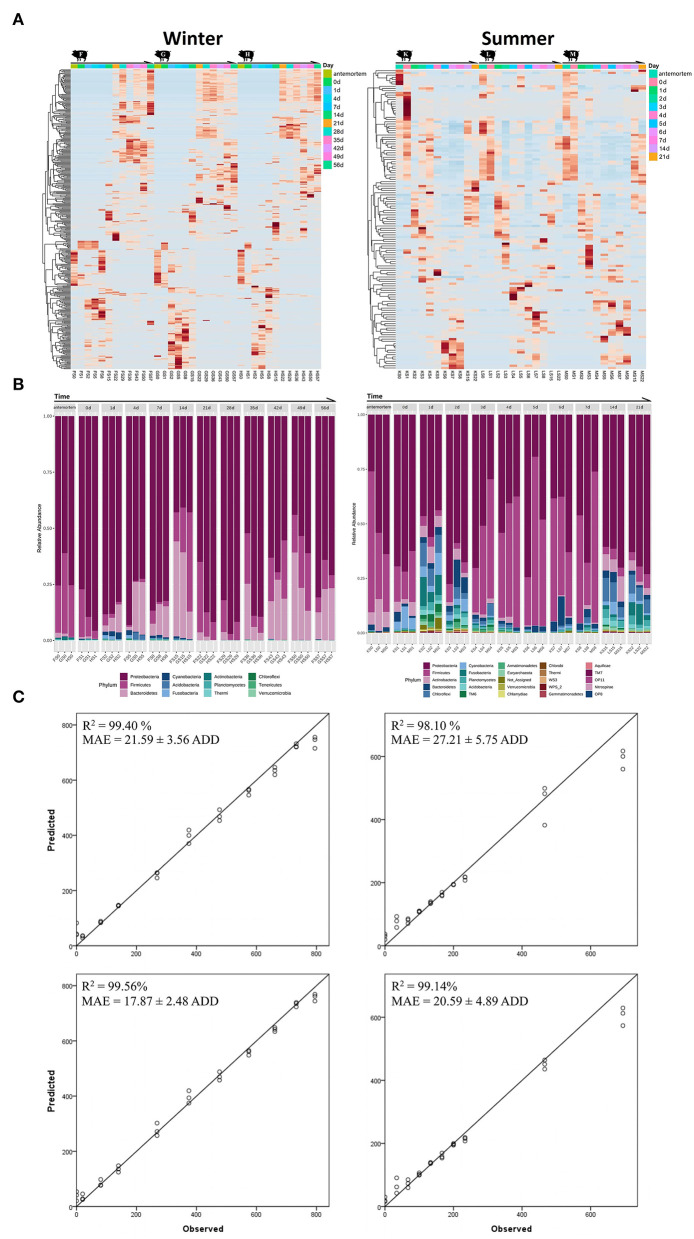
The heat maps, taxa histograms, and RF models for the winter (left panel) and summer (right panel) trials. **(A)** Heat maps for the epinecrotic communities of both seasons. The samples on the *X*-axis are grouped by repeating experimental groups and ordered by the sampling time. The OTUs on the *Y*-axis are hierarchically clustered with the Minkowski distance matrices. **(B)** The phylum composition and variation of the epinecrotic communities during both seasons. The samples are arranged chronologically on the *X*-axis. The sample time points are marked on the top of each stacked bar and “a” represents the antemortem. The stacked bars of disparate colors show the relative abundance of each phylum in the epinecrotic communities. **(C)** The predicted ADDs calculated by the established RF models vs. observed ADDs in each trial were plotted with a one-to-one line added for reference. The RF models are based on the total OTUs (the top two plots) and biomarker sets (the bottom two plots) illustrate the correlation between observed ADD and predicted ADD.

The temporal successions of the epinecrotic communities are shown in the heat maps by hierarchically clustering OTUs with Minkowski distance matrices (Figure 3A). Remarkable and repeatable succession patterns have been observed in the epinecrotic biofilms. Significant clusters could been found in the heat map for the winter trial. The features changed significantly across the time points of 10 min before the sacrifice, immediately after the sacrifice, and during the subsequent decay process in the water, especially during the 2–3 weeks when the carcasses were in the advanced floating decay stage. For the summer trial, the epinecrotic community structure changed significantly on the first day in the water and then showed a sequential succession pattern during the subsequent decay process (2–7 days). Additionally, the main classes of the 14 and 21 days which are specific to the communities on the bone remains were similar to the cluster which represented the early decay stage. In the epilithic and aquatic communities, the clustered 16S OTUs with high abundance changed significantly during the winter trial. However, both communities in summer were short of variable OTUs, and performed more like the macro-succession pattern (Supplementary Figure S9).

Taxa annotation and abundance variation for chronological samples are shown as histograms in Figure 3B. For the winter trial, the phyla of Proteobacteria (79.04 ± 11.09%) and Firmicutes (18.91 ± 10.44%) have been the dominant taxa before the body have entered the water (antemortem and immediately after being sacrificed). During the subsequent decay process in the water, Proteobacteria was still dominant (72.83 ± 17.04%, 1–56 days), whereas Firmicutes had decreased through the submerged fresh stage to the floating decay stage (4.44 ± 5.59%, 0–7 days) and then followed to slowly increase through the advanced floating decay stage to sunken remains stage (15.55 ± 10.35%, 14–56 days). In addition, Bacteroidetes which represented a minor part of the antemortem communities (1.26 ± 0.81%) had increased gradually through the submerged fresh stage to the floating decay stage (16.20 ± 12.61%, 1–14 days). At the advanced submerged decay stage, Bacteroidetes had first decreased (1.54 ± 1.53%, 21–28 days) and then increased up until the sunken remains stage (19.18 ± 9.39%, 35–56 days). Other phyla, such as Cyanobacteria, Acidobacteria, and Fusobacteria were scarce and occurred opportunistically during the winter trial. For the summer trial, the phyla of Proteobacteria (57.95 ± 15.39%) and Firmicutes (28.93 ± 17.03%) were the dominant taxa before the body entered the water. During the subsequent decay process in the water, Proteobacteria was still dominant (53.73 ± 15.62%, 1–21 days), whereas Firmicutes had decreased in the early floating stage (4.54 ± 1.09%, 1–2 days), and then became substantially more abundant during the floating decay stage and advanced floating decay stage (45.56 ± 16.80%, 3–7 days). On the bone remains (14 and 22 days), Firmicutes only accounted for 4.92 ± 1.33% of the communities. Other phyla, such as Acidobacteria, Bacteroidetes, and Chloroflexi, which were abundant right after the carcasses have entered the water diminished during the decay process, but bacame abundant on the bone remains. The variations of the top 40 genera of the epinecrotic communities are shown in Supplementary Figure S10.

### PMSI Estimation Models Based on the Epinecrotic Biofilm Succession

We regressed the relative abundances of total OTUs against ADDs using the RF machine learning algorithm to establish the models to predict PMSI based on the epinecrotic biofilm succession. The models based on the data of the winter and summer trials explained 98.90 and 95.80%, respectively, of the ADDs since the placement of carcasses in the water. Figure 3C depicts the predicted ADDs vs. the observed ADDs, with the one-to-one line added for reference. The *R*^2^ values between observed and predicted ADDs were 99.40 and 98.10%, and the MAEs were 21.59 ± 3.56 and 27.21 ± 5.75 ADD, respectively. Since the average daily water temperature in the non-flowing aquatic environment was mostly stable during the winter and summer trials (14.25 ± 3.70 and 32.51 ± 1.22°C, respectively), ADDs increased linearly during both seasons, which allowed the models to predict PMSI with the error about 1.5 and 0.8 days during 56 and 21 days of decomposition, respectively.

A large proportion of OTUs in the epinecrotic biofilm did not affect PMSI prediction. We list the taxa annotation of the top 167 and 186 important OTUs which made significant contributions (≥0.1%) to the accuracy of the RF models, respectively (Supplementary Table S5). It should be noted that these influential taxa use for the PMSI estimation were not present at the higher abundances in the epinecrotic biofilm. They only accounted for 36.12 ± 15.60 and 18.25 ± 12.50% of the communities in each season. For establishing more efficient models, the OTUs with low contribution were removed and the important OTUs were used as the predictive biomarkers in the training sets of the RF machine learning algorithm. In comparison to using total OTUs, the *R*^2^ values of the RF models based on the biomarker sets increased to 99.56 and 99.14%, and the MAEs decreased to 17.87 ± 2.48 and 20.59 ± 4.89 ADD, respectively (Figure 3C). It means that the models allow us to predict PMSI with the error of about 1.3 and 0.7 days during 56 and 21 days of decomposition, respectively. Since only 10 biomarkers were shared in the training data of the winter and summer trials, the cross-validation of the different season models was weak in *R*^2^ values (0.20 and 0.69%) and MAEs (202.73 ± 29.57 and 292.25 ± 33.95 ADD). These findings suggested that the RF models based on different seasons cannot be interchangeably used for PMSI prediction.

## Discussion

While there have been several studies of the PMI estimation using the microbial succession related to carcasses in the terrestrial ecosystems (Guo et al., 2016; Fu et al., 2019), research of such scope is scarce for the aquatic environments. In the present study, the bacterial succession patterns of epinecrotic and epilithic biofilms in a non-flowing aquatic habitat have been analyzed by sequencing the V4 region of 16S rDNA. Similar to the findings of the previous terrestrial carcass decomposition studies, prominent bacteria successions occurred in a predictable and reproducible manner on the surfaces of the carcasses submerged in water. As shown in the heat maps (Figure 3A), the clusters of high abundance taxa changed across each of the decay stages. In addition, these successive changes were highly repetitive between carcasses within coincident experimental settings. In the human cadaver experiments done by Metcalf et al. (2016), the prominent successions of bacterial communities across terrestrial subjects within a season have been observed. The heat maps which were generated with a similar approach as ours have shown the reproducible succession patterns of bacterial communities colonizing the skin of human cadavers. The conclusions of these studies indicated that the epinecrotic community structure variations during the postmortem decomposition processes of terrestrial and aquatic cadavers followed specific succession patterns, which could be quantitatively analyzed by HTS and potentially applied for estimating PMI/PMSI. However, the disparate community structures and succession patterns in different seasons have been observed (Figures 3A,B; Supplementary Figure S10), meaning that existing findings were not sufficient for the use in diverse environments. Whether this bacteria-dependent method can be used in the forensic practice depends on the determination of the influence of various environmental factors on the succession patterns. Through the present study, we can confirm that the seasonal conditions, such as temperature, light, and water quality, can be the critical factors to community successions and need to be further clarified in the subsequent researches, which should be conducted in the artificially controlled conditions.

The epinecrotic communities of aquatic and terrestrial carcasses shared similar dominant bacterial phyla (Proteobacteria, Firmicutes, Bacteroidetes, and Acidobacteria). Using the replicate swine carcasses as models for the human decomposition research, Pechal et al. (2014) studied the epinecrotic bacteria throughout decomposition in the forest habitat. Each decay stage had a unique profile of four dominant phyla that were changing in disparate trends. Proteobacteria was the most dominant phylum which decreased over time. Firmicutes became the dominant taxon as decomposition progressed. Bacteroidetes occurred only in the fresh and bloated stages. Actinobacteria, which represented the majority of the communities found on the skin of the carcasses, had disappeared during the dry stage. In the present study of the drowned swine carcasses (Figure 3B), Proteobacteria and Firmicutes were the dominant phyla before the body had entered the water. During the decay stages in the water, Proteobacteria remained dominant throughout, while. Firmicutes had first decreased, and then increased in the floating decay stage. Bacteroidetes representing a minor part of the antemortem communities had increased first, followed with the short decrease, and then kept increasing up until the sunken remains stage. Acidobacteria was scarce and occurred opportunistically throughout the decomposition. It can be inferred from the above results that the taxonomic compositions of cadaver epinecrotic communities were similar, whereas the variation trends of each phylum were completely different between the habitats. This may be one of the main reasons why cadaver decomposition is significantly faster in the terrestrial setting (5 days) than that in the water (14 days in summer and 56 days in the winter). Therefore, it is necessary to further study the epinecrotic communities on the aquatic cadavers to widen their extent for the forensic microbiology application.

To date, there have been several studies on the PMSI estimation using succession patterns of the microbial communities on the aquatic carcasses, involving prokaryotic and eukaryotic microflorae. Hyun et al. investigated microeukaryotic biodiversity and community structures on the drowned pig (Hyun et al., 2019). The sequencing analysis showed the water molds and algae were related to the carcass decomposition. Relative abundances of the *Filobasidium, Achlya, Saprolegnia, Hydrodictyon, Lobosphaera*, and *Scenedesmus* varied across the decay stages. However, the change in microeukaryotic biodiversity with the decomposition progression was not significantly related to the PMSI. The insufficient biological replication limited the establishment of a mathematical model for the PMSI estimation. By using numerous fresh porcine cadaver bones as the biological replications, Randall et al. determined that eukaryotic community succession had occurred on porcine skeletal remains in a freshwater lake, which allowed the development of the mathematical approach for the PMSI estimation using the RF regression (Randall et al., 2021). Resulting models for the sample data from the ribs and scapulae predicted PMSI with errors of ± 104 (937 ADD) and ± 63 days (564 ADD), respectively. Such high error rates for PMSI estimation suggested that the eukaryotic succession patterns may not be applicable for the rigorous forensic investigation. Notably, the existing body of research on microbial succession on aquatic carcasses was focused mostly on bacterial microflora, especially on that in the epinecrotic biofilms formed on the liquid–solid surfaces. Recently, Cartozzo et al. (2021) have conducted a parallel experiment with Randall et al. (2021), which also used porcine bones submerged in the similar aquatic habitat to explore the temporal changes in the bacterial community structure. They found that the community alpha diversity increased with ADD. Similarly, beta diversity changed significantly with ADD and had been reasonably explained using the environmental parameters and inferred functional pathways. RF models developed using the 24 ribs and 34 scapula family level taxa allowed the prediction of PMSI with root mean square error (RMSE) of 57 (522.97 ADD) and 37 days (333.8 ADD), respectively. By comparing the above parallel experiments, it is apparent that the PMSI could be estimated more accurately from the bacterial succession patterns than that from eukaryotes. Instead of using the porcine bones, present study explored the bacterial succession on the surfaces of intact swine carcasses. Based on the RF models, epinecrotic communities in winter and summer were determined to estimate PMSI with MAEs of 1.3 (17.87 ± 2.48 ADD) and 0.7 days (20.59 ± 4.89 ADD). Apparently, these results can so far only represent the application of epinecrotic biofilm in the present specific habitats. We also find that the established models based on different seasons cannot be interchangeably used due to the rarely shared taxon. Whether it can be applied to other environments remains to be further explored by studying the mechanisms of influence of the environmental factors (e.g., water quality parameters, and aquatic microbiota) affecting the bacterial succession.

The succession patterns of bacterial communities found in the present study have a certain degree of discrepancy from similar studies conducted in other aquatic habitats. Previously, we conducted the experiment on the rat carcasses in the water containers to control the environmental variables such as water temperature and DO (He et al., 2019). Consistent with the present study, the phyla of Proteobacteria, Bacteroidetes, Firmicutes, and Actinobacteria were abundant in the rat epinecrotic communities. However, the abundance of the Proteobacteria decreased while Firmicutes increased during decomposition in the past, which partially contradicted the results of the present experiments conducted in the field ponds (Figure 3B). Similar to the present experimental settings, Benbow et al. (2015) have described the bacterial community succession in the epinecrotic biofilms of swine carcasses in a flowing stream during summer and winter. They also confirmed that Proteobacteria decreased as Firmicutes increased over the decomposition process during both seasons, which remained the different from our experiments conducted in the non-flowing ponds (Figure 3B). In fact, the variation trends of Proteobacteria and Firmicutes in our observation seemed much more complicated. They did not show continuous growth or a downward trend, but rather fluctuated across decay stages. For example, in the summer trial, Firmicutes increased significantly from the floating decay stage (3 days) to the sunken remains stage (7 days), while Proteobacteria had just a slight decrease. However, Firmicutes was low in abundance on the final skeleton remains (14 & 21 days), with an increase of Proteobacteria (Figure 3B). In a study that most closely matched our experimental settings and conditions, Kaszubinski et al. (2020) detected the bacterial community structures on the surfaces of three replicate swine carcasses submerged in a non-flowing pond over six time points (from submerged fresh to advanced floating decay stages) in summer. They found that Firmicutes increased and then decreased with Proteobacteria changing reversely over the decomposition. The most accurate model for PMSI estimation was a quadratic regression of phyla Firmicutes, Proteobacteria, and Bacteroidetes. Compared with the previous studies, more sampling time points (11 in our summer trial) gave us a more detailed interpretation of the epinecrotic community succession in the aquatic habitats. However, the discrepant and discontinuous variations in the taxa abundance that occurred in different habitats also posed a challenge for establishing mathematical model for the PMSI estimation.

In addition, it can be concluded from our results that although the decomposition had progressed in the same habitats, there were prominent differences between the succession patterns that occurred during the summer and winter. The water temperature, DO, conductivity, and salinity in the summer were significantly higher than those in winter (Supplementary Figure S1), which could produce variations in the aquatic bacterioplankton. In the present study, the species richness of the aquatic communities in winter was significantly lower than that in summer (Supplementary Figure S4). A great discrepancy in the community diversity between the two seasons is illustrated in the Supplementary Figure S8. There were no significant differences of the water quality and community diversity between the locations whether the carcasses had presented (Supplementary Figure S6). The above information provides the implication that the effect of the seasonal factors on the bacterioplankton community was greater than that of the presence of the decomposing carcasses. In this case, the succession patterns of the epinecrotic community are different between seasons but repeatable in the parallel experimental settings (Figures 3A,B), correlating with the carcasses in summer decomposing much faster than those in winter, while having the equal decomposition rates during the identical season. The seasonal differences in the epinecrotic bacterium have also been demonstrated in the research of Dickson et al. (2011) and Benbow et al. (2015). They suggested that seasonal factors influence bacterial composition more than the decomposition process itself. Understanding the seasonal influences and other environmental factors on aquatic and epinecrotic communities is essential to accurately estimate the PMSI, especially for the long-term decomposition process across the different seasons.

In the present study we have mainly focused on the bacterial successions in the biofilm formed on the liquid–solid surfaces. The formation and maturation of the biofilm on different substrates were accompanied by the specific bacterial succession patterns. Several differential taxa between the epinecrotic and epilithic communities have been identified by LEfSe analysis (Supplementary Tables S3, S4). Significant different succession patterns have been observed (Figure 3A; Supplementary Figure S9). The alpha diversity on carcasses was lower than that on tiles (Supplementary Figure S5). The distances between the two sample types increased with the PMSI (Figure 2A). Similar to the aquatic communities during both trials, the epilithic samples clustered stably. The epinecrotic communities showed significant variations through time, and while being initially close to the epilithic samples, subsequently separated from the other dots on the PCoA plots. These results were consistent with our previous experiment done in the water containers (He et al., 2019). Several studies have already demonstrated that epilithic and epixylic (decaying plant material) biofilms differ in community composition, limiting nutrients, exoenzyme activity, and fungal biomass (Das et al., 2007; Sinsabaugh et al., 2010; Tank and Dodds, 2010). Lang et al. (2016) further found significant differences in the community composition between the two biofilm types (inorganic vs. carrion). Notably, dominating microorganisms in the epinecrotic community included a large portion of heterotrophs or detritivores, whereas the epilithic community was mostly represented by autotrophs. This point of view has been further testified by Hyun et al. (2019), who found that the microeukaryotic communities on carcasses were also significantly different from those in the abiotic control objects. Considering the influences of seasonal and other environmental factors, the succession patterns of epilithic biofilm which coexist with epinecrotic biofilm in most aquatic habitats can be used as a temporal control for PMSI estimation.

In conclusion, this study explored the bacterial community succession patterns associated with the vertebrate remains decomposing in the water for further use in the PMSI estimation. We successfully identified the epinecrotic and epilithic bacterial community structure variations throughout the decomposition processes of carcasses submerged in the non-flowing ponds during two seasons, providing preliminary support for potential use of the biofilm communities in the forensic investigations. The prominent bacterial succession patterns in the epinecrotic biofilms formed on the solid–liquid surfaces of the aquatic carcasses provide a new insight for the accurate PMSI estimation. The influencing mechanisms of the environmental factors and the aquatic bacterioplankton on the epinecrotic biofilm communities should be studied further before the biofilm could be considered as an accurate indicator of the PMSI.

## Data Availability Statement

The datasets presented in this study can be found in online repositories. The names of the repository/repositories and accession number(s) can be found in the article/Supplementary Material.

## Ethics Statement

The animal study was reviewed and approved by the Medical Ethics Committee of Xiangya Hospital, Central South University.

## Author Contributions

XFu and JC designed the study. YH, YL, and XFa collected the samples for all analyses. LZ performed DNA extraction for 16S rDNA sequencing. KJ performed raw data analysis. FD and JG performed the statistical analysis. All authors contributed, reviewed, and approved the manuscript.

## Funding

This work was funded by the National Natural Science Foundation of China (81971791 and 82030058), Scientific Research Fund of Zhejiang Provincial Education Department (Y202146041), Basic Research Funds of Hangzhou Medical College (KYYB202009), Doctoral Scientific Research Foundation of Hangzhou Medical College (00004F1RCYJ2001), and Undergraduate Training Program for Innovation of Zhejiang Province (S202113023095 and S202113023093).

## Conflict of Interest

The authors declare that the research was conducted in the absence of any commercial or financial relationships that could be construed as a potential conflict of interest.

## Publisher's Note

All claims expressed in this article are solely those of the authors and do not necessarily represent those of their affiliated organizations, or those of the publisher, the editors and the reviewers. Any product that may be evaluated in this article, or claim that may be made by its manufacturer, is not guaranteed or endorsed by the publisher.
