# Supplementary material for: Bacterial Succession in Microbial Biofilm as a Potential Indicator for Postmortem Submersion Interval Estimation
Source: Front Microbiol. 2022 Jul 22;13:951707. doi: 10.3389/fmicb.2022.951707 (PMC9356301; doi:10.3389/fmicb.2022.951707)
Supplement: Supplementary file 2 [file Data_Sheet_2.pdf]

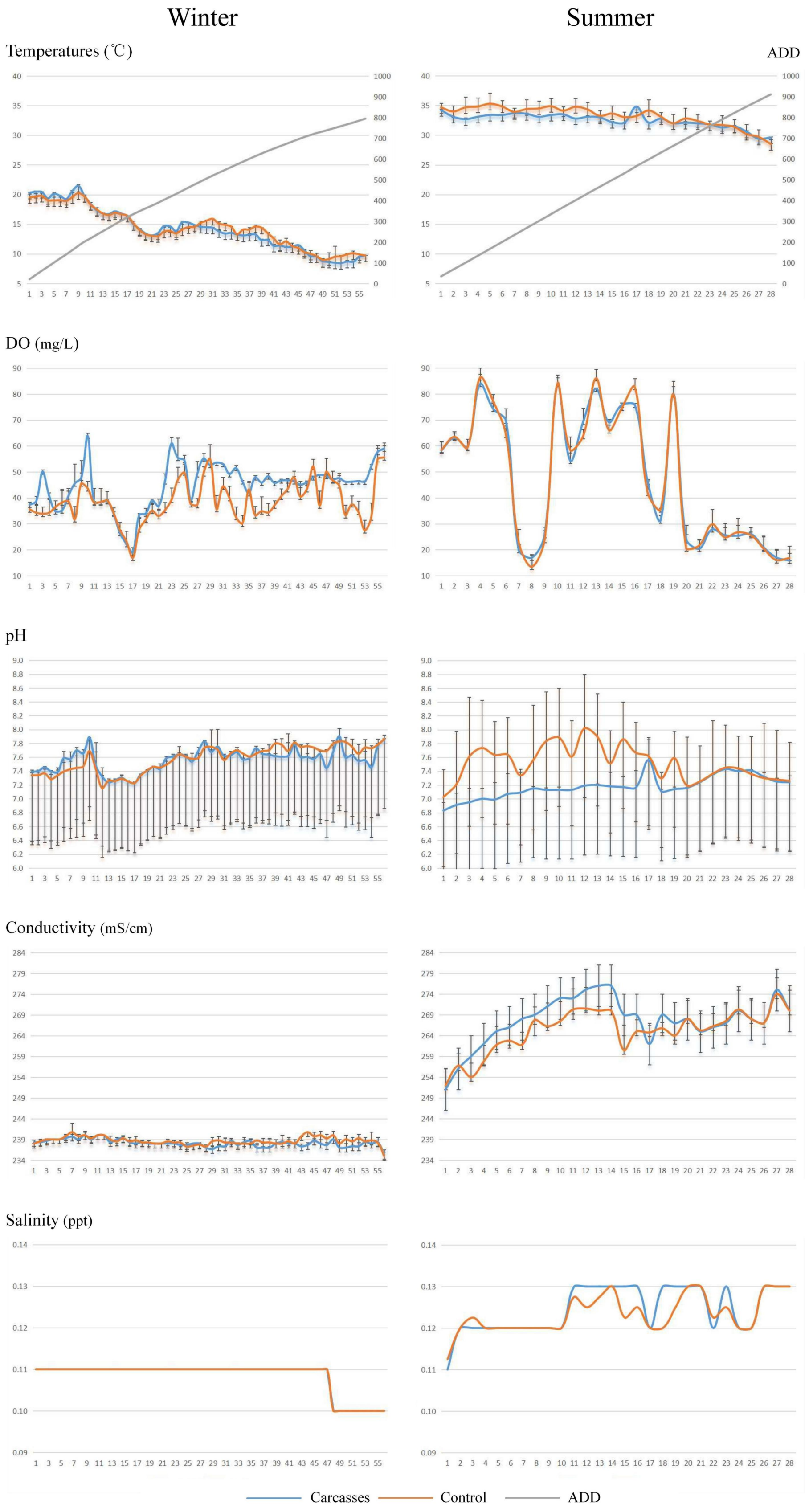

Figure S1. The water quality parameters measured at the locations for sunken carcasses and negative control both in the winter and summer trials. The X-axis of each plot is the number of days since the carcasses entered the water.

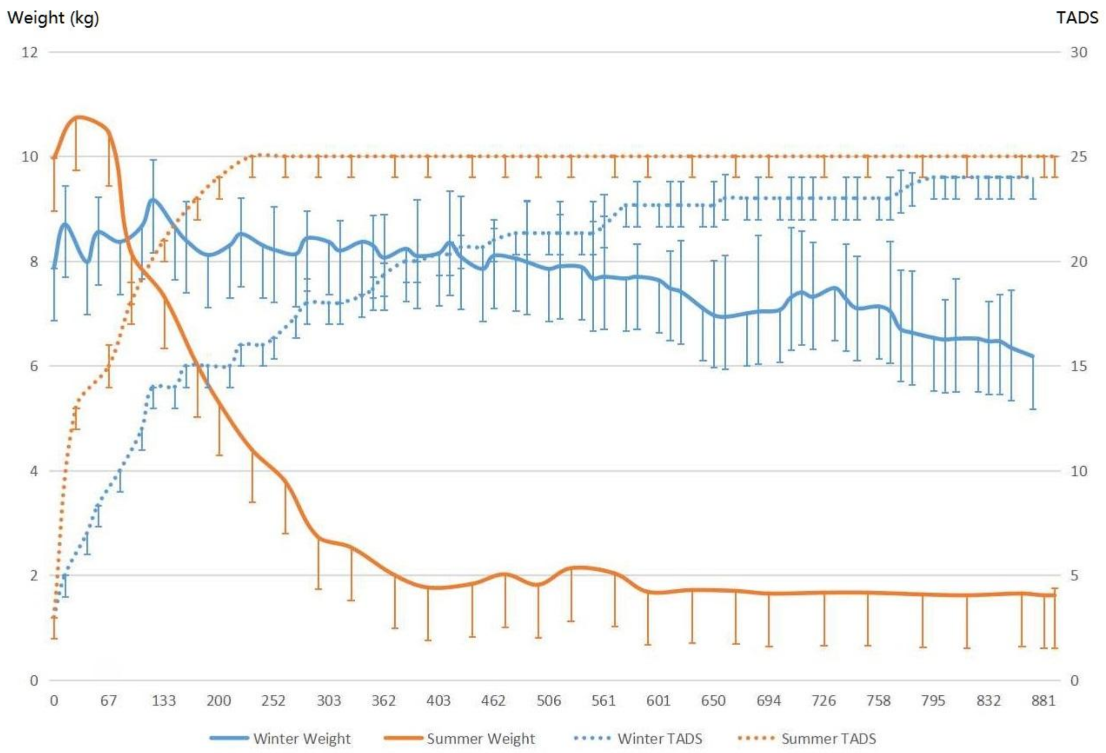

Figure S2. The body weight and TADS score variation during the decomposition processes since the carcasses entered the water. The X-axis of the plot is the value of ADD.

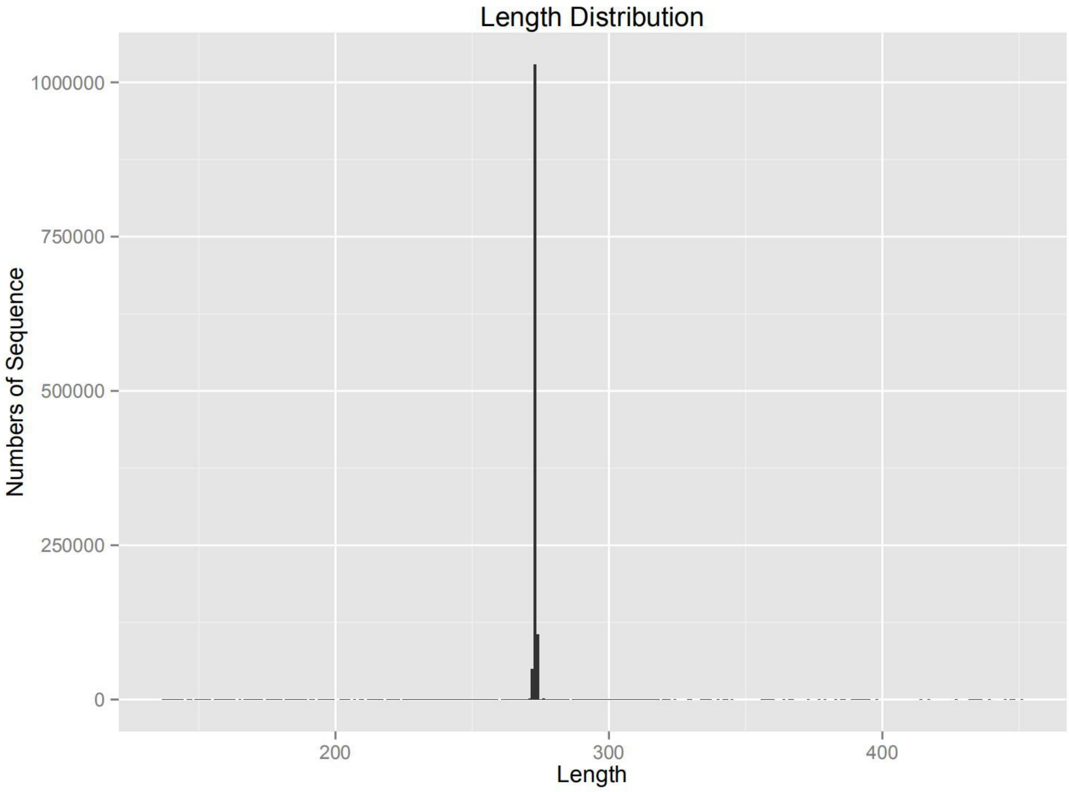

Figure S3. The length distribution of the reads determined by sequencing were rang in 200~300bp, which fits the size of 16S V4 region.

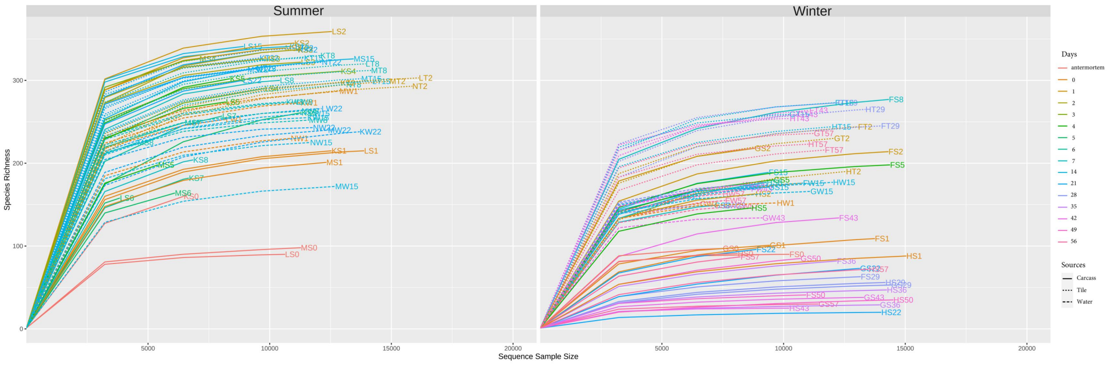

Figure S4. The rarefaction curves for the sequencing samples obtained from winter and summer trials. All the curves had approached the plateau phases.

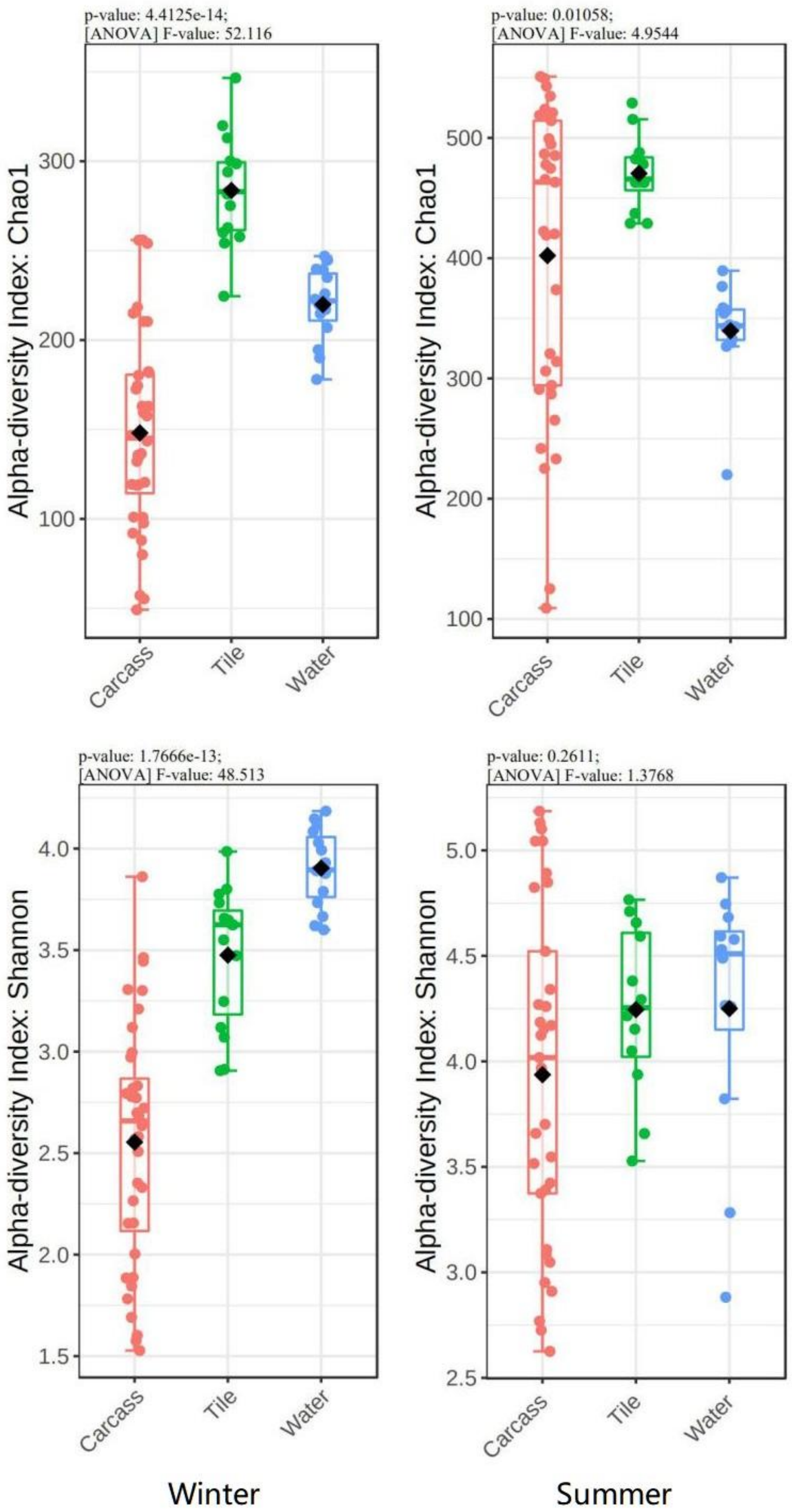

Figure S5. Comparison of alpha diversity between epinecrotic (carcass), epilithic (tile) and aquatic (water) communities. Each sample was measured with Chao1 and Shannon indices, as shown in each box plot. The ANOVA results are showing at the top of each plot.

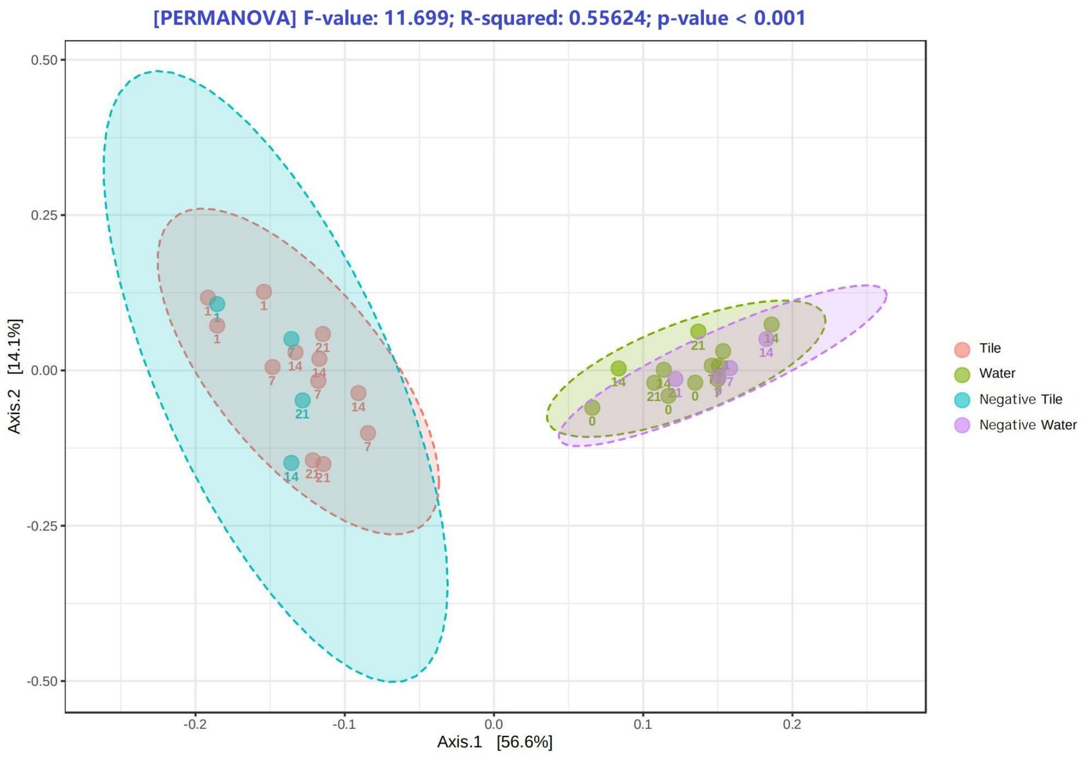

Figure S6. Two-dimensional PCoA plots of weighted Unifrac distance matrices for epilithic (tile) and aquatic (water) samples obtained from summer trial and negative control. The statistical significance of the clustering pattern in each plot was evaluated with PERMANOVA (top). Samples obtained from tiles and waters are presented in different colors. Sampling time points are marked under each dot.

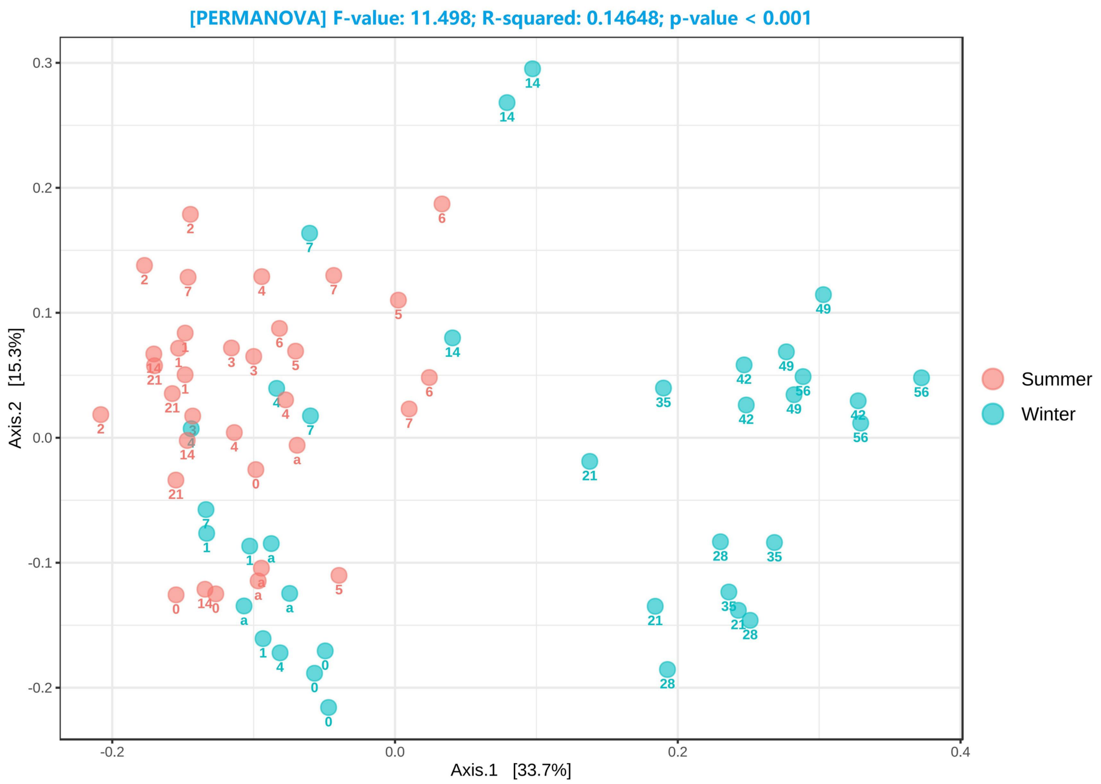

Figure S7. Two-dimensional PCoA plots of weighted Unifrac distance matrices for epinecrotic samples (carcasses) obtained from summer and winter trials. The statistical significance of the clustering pattern in each plot was evaluated with PERMANOVA (top). Samples obtained from summer and winter trials are presented in different colors. Sampling time points are marked under each dot and “a” represents antemortem time point.

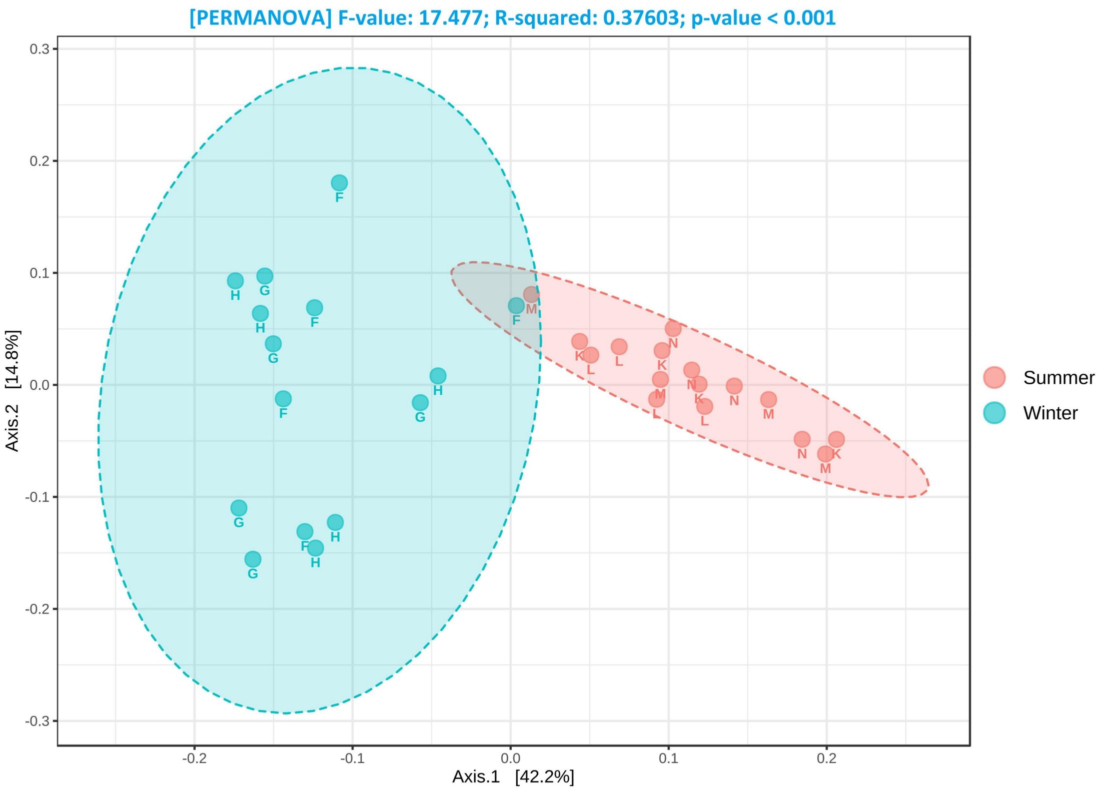

Figure S8. Two-dimensional PCoA plots of weighted Unifrac distance matrices for aquatic samples (water) obtained from summer and winter trials. The statistical significance of the clustering pattern in each plot was evaluated with PERMANOVA (top). Samples obtained from summer and winter trials are presented in different colors. Sampling locations are marked under each dot. “N” for negative controls of water samples without presence of carcasses, “K, L, M” for samples obtained in the summer trial and “F, G, H” for samples obtained in the winter trial.

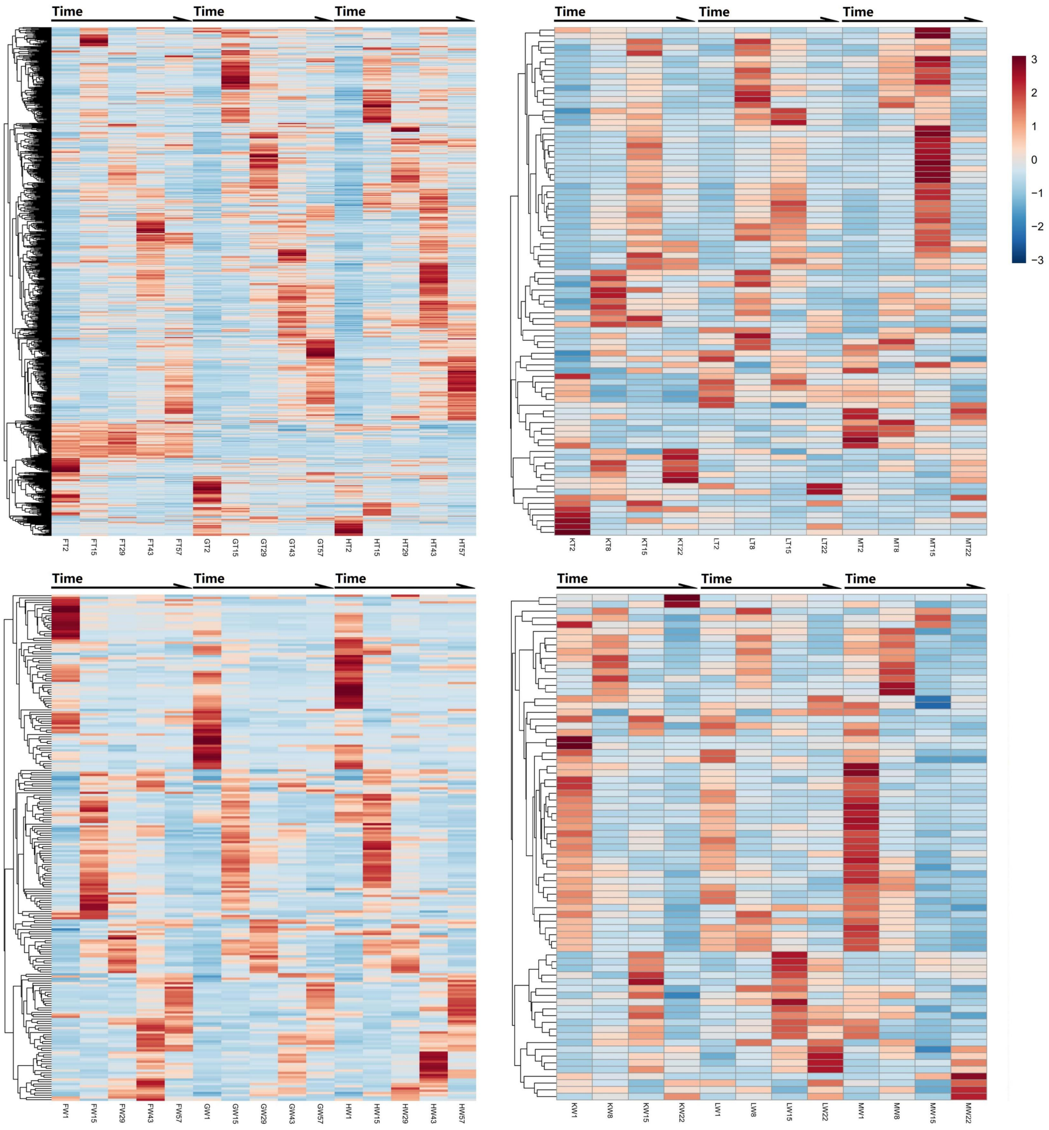

Figure S9. Heat maps for epilithic (above) and aquatic (below) communities of winter (left) and summer (right) trials. The samples on the X-axis are grouped by repeating experimental groups and ordered by sampling time. The OTUs on the Y-axis are hierarchically clustered with the Minkowski distance matrices.

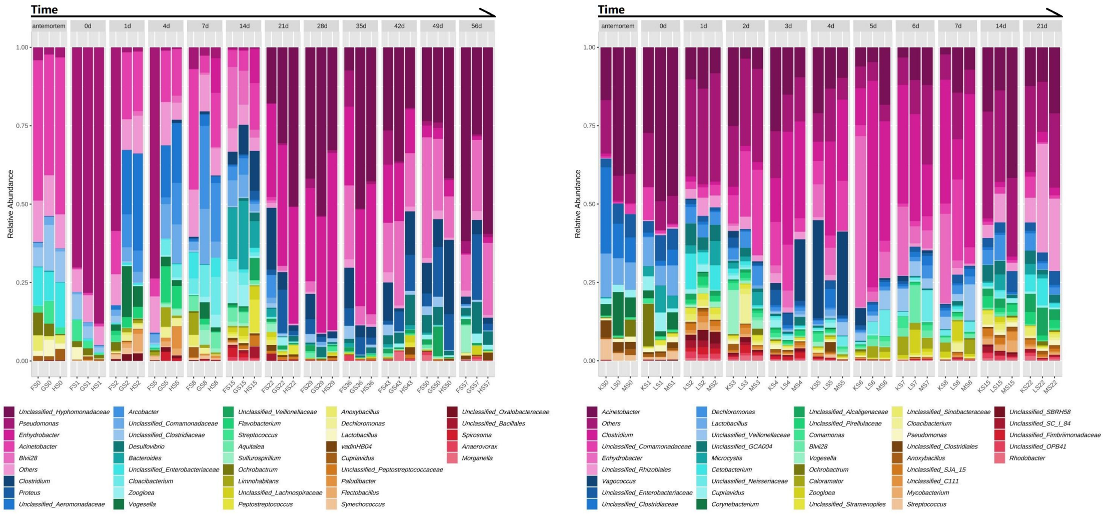

Figure S10. The taxonomic composition and variation of the epinecrotic communities in winter (left) and summer (right) trials. The samples are arranged chronologically on the X-axis. The sample days are marked on the top of each stacked bar and "a" represents antemortem time point. The stacked bars show the relative abundance of top 40 genera in the epinecrotic communities.
